# Supplementary material for: First Insights into Body Localization of an Osmoregulation-Related Cotransporter in Estuarine Annelids
Source: Biology (Basel). 2024 Mar 31;13(4):235. doi: 10.3390/biology13040235 (PMC11048583; doi:10.3390/biology13040235)
Supplement: Supplementary file 1 [file biology-13-00235-s001.zip › Supplementary Materials/SupplementaryFigures S1-S7.pptx]

## Slide 1
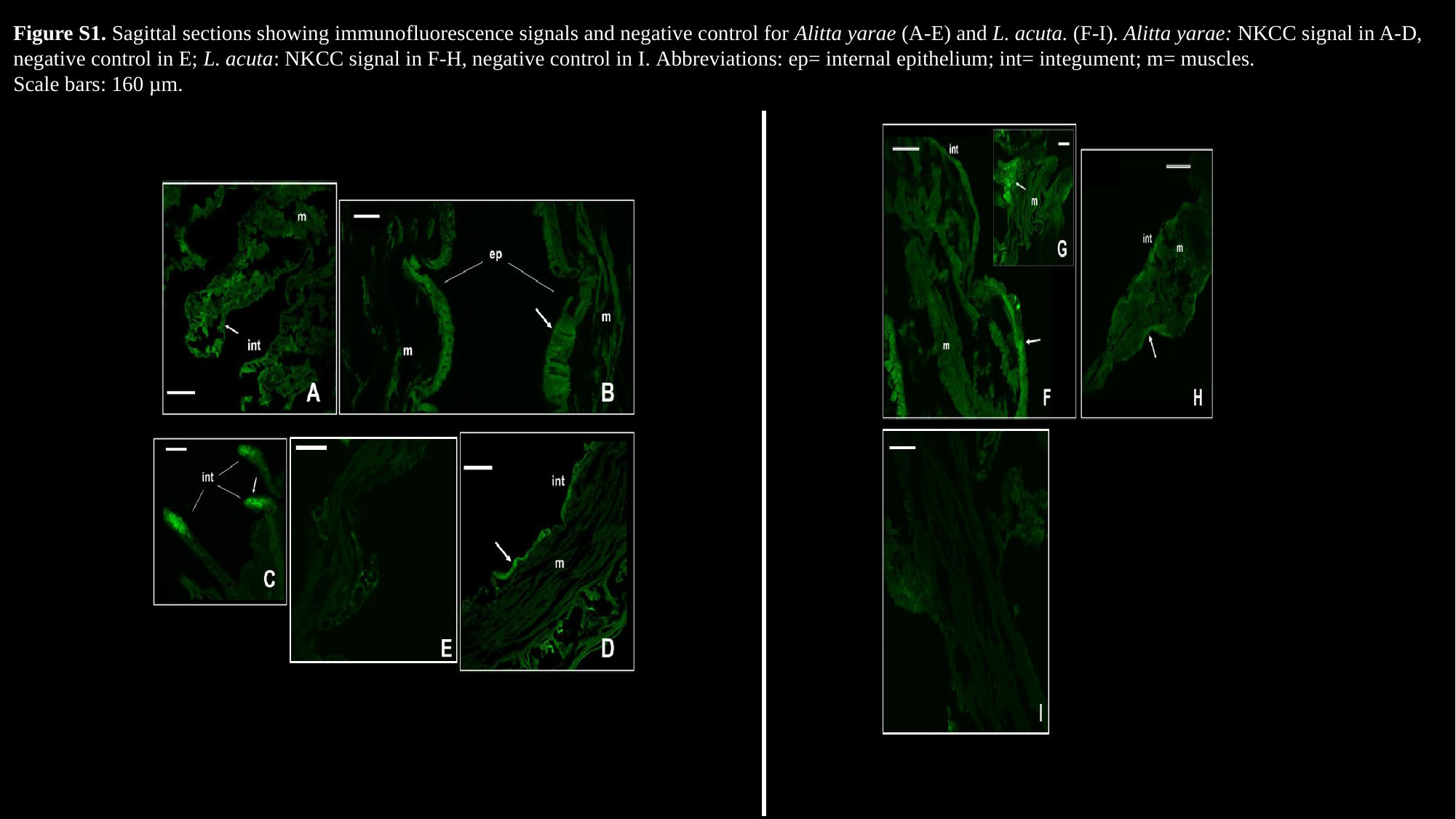

Figure S1. Sagittal sections showing immunofluorescence signals and negative control for Alitta yarae (A-E) and L. acuta. (F-I). Alitta yarae: NKCC signal in A-D, negative control in E; L. acuta: NKCC signal in F-H, negative control in I. Abbreviations: ep= internal epithelium; int= integument; m= muscles.
Scale bars: 160 µm.

## Slide 2
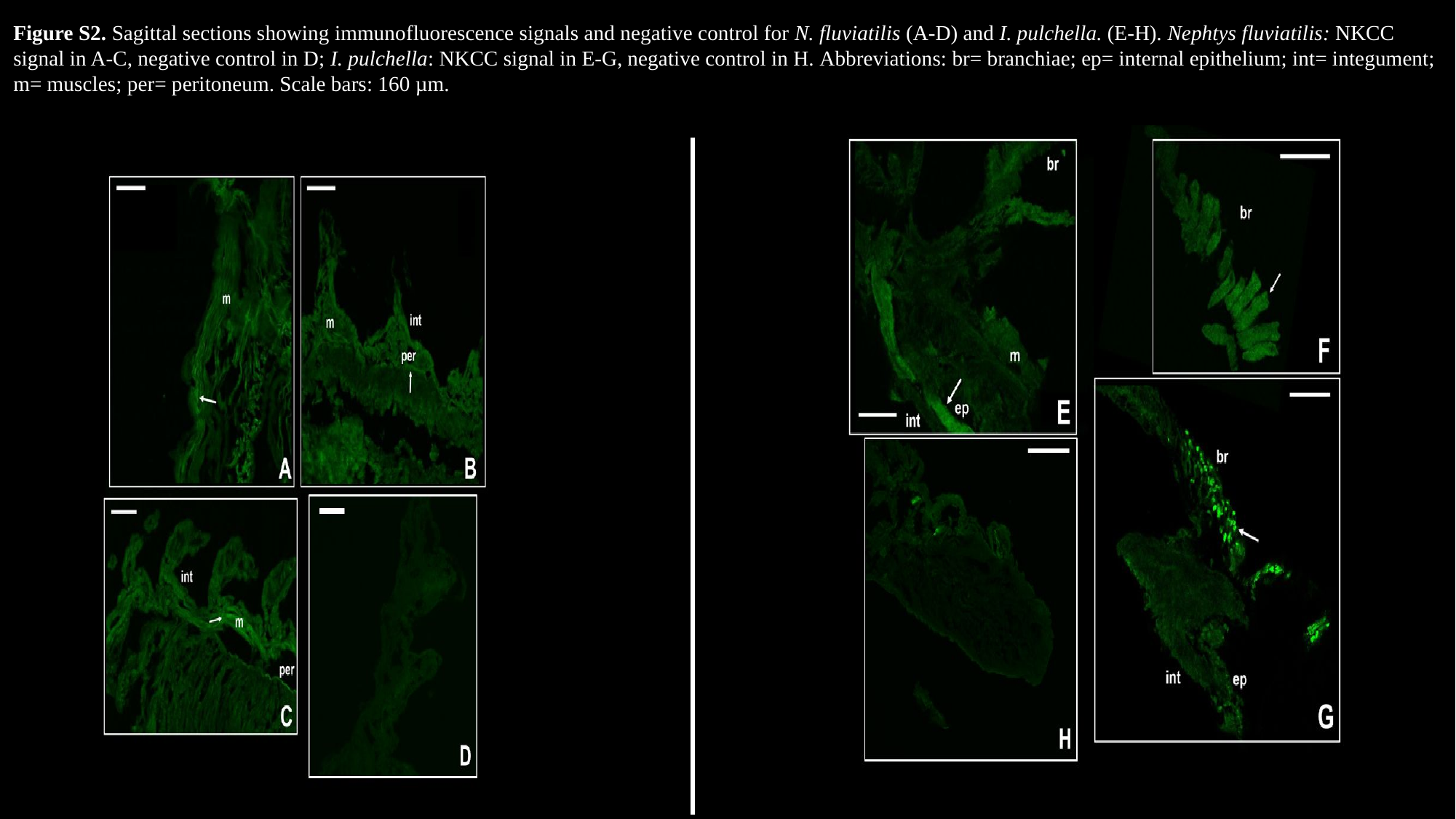

Figure S2. Sagittal sections showing immunofluorescence signals and negative control for N. fluviatilis (A-D) and I. pulchella. (E-H). Nephtys fluviatilis: NKCC signal in A-C, negative control in D; I. pulchella: NKCC signal in E-G, negative control in H. Abbreviations: br= branchiae; ep= internal epithelium; int= integument; m= muscles; per= peritoneum. Scale bars: 160 µm.

## Slide 3
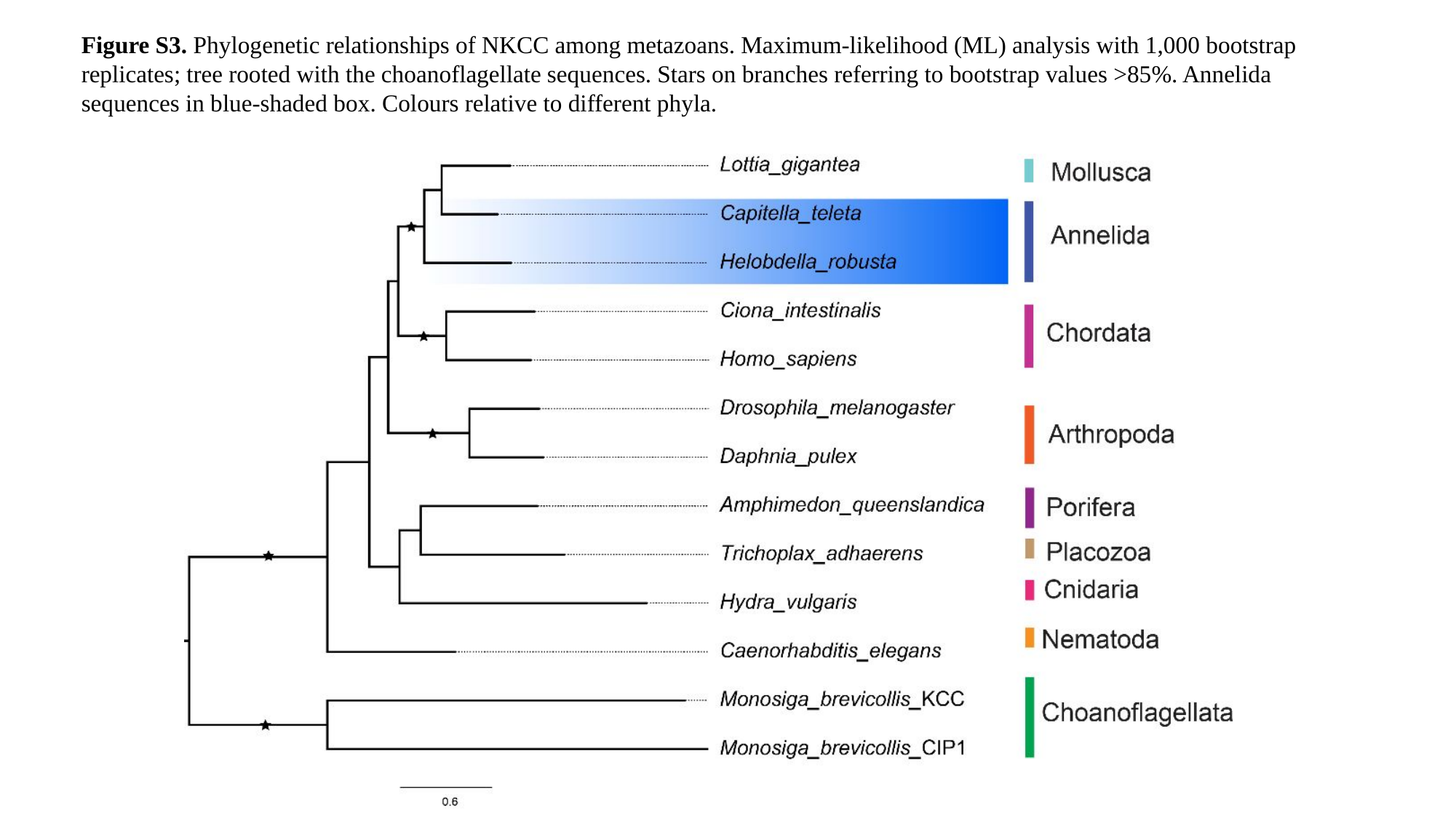

Figure S3. Phylogenetic relationships of NKCC among metazoans. Maximum-likelihood (ML) analysis with 1,000 bootstrap replicates; tree rooted with the choanoflagellate sequences. Stars on branches referring to bootstrap values >85%. Annelida sequences in blue-shaded box. Colours relative to different phyla.

## Slide 4
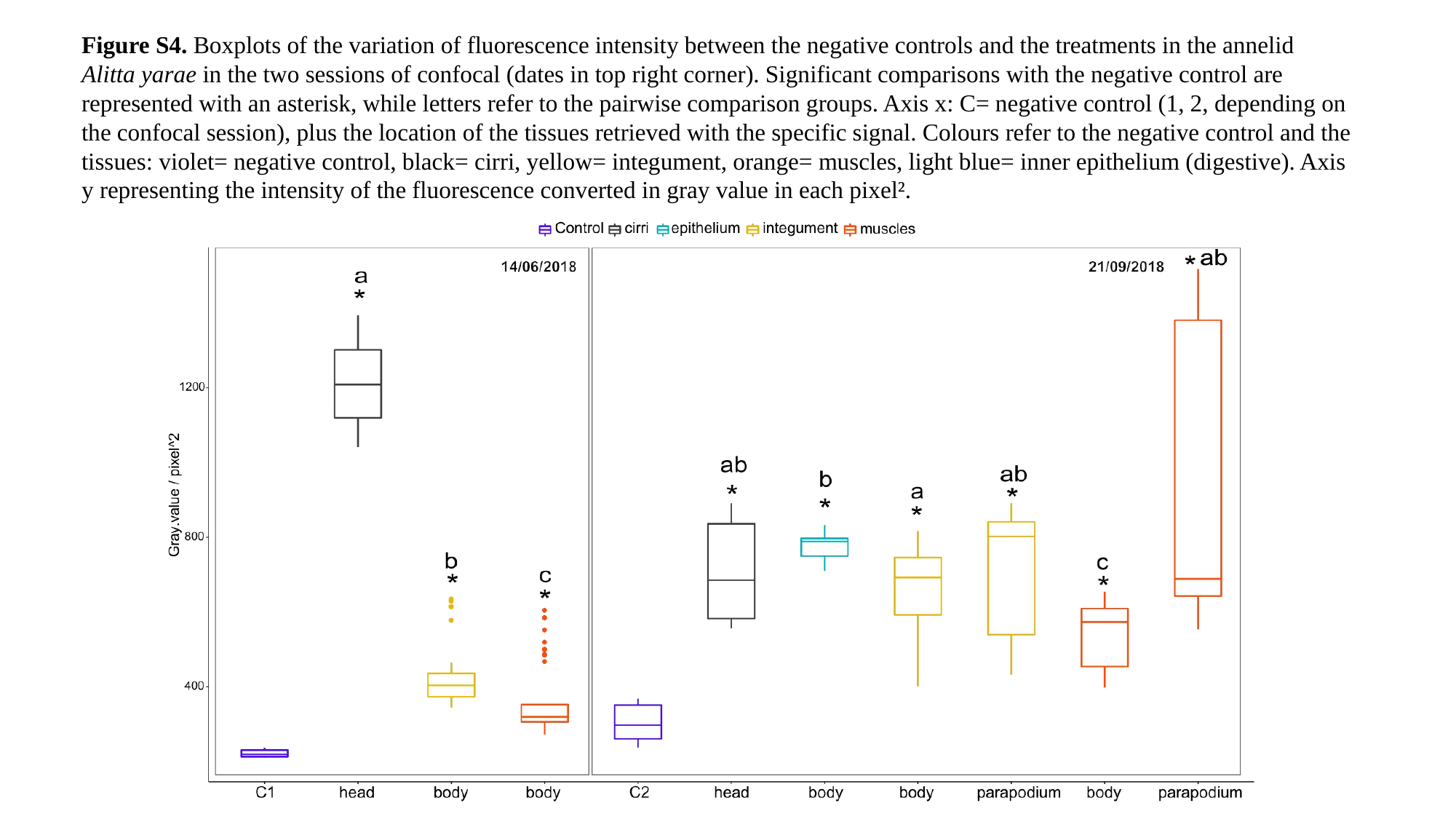

Figure S4. Boxplots of the variation of fluorescence intensity between the negative controls and the treatments in the annelid Alitta yarae in the two sessions of confocal (dates in top right corner). Significant comparisons with the negative control are represented with an asterisk, while letters refer to the pairwise comparison groups. Axis x: C= negative control (1, 2, depending on the confocal session), plus the location of the tissues retrieved with the specific signal. Colours refer to the negative control and the tissues: violet= negative control, black= cirri, yellow= integument, orange= muscles, light blue= inner epithelium (digestive). Axis y representing the intensity of the fluorescence converted in gray value in each pixel².

## Slide 5
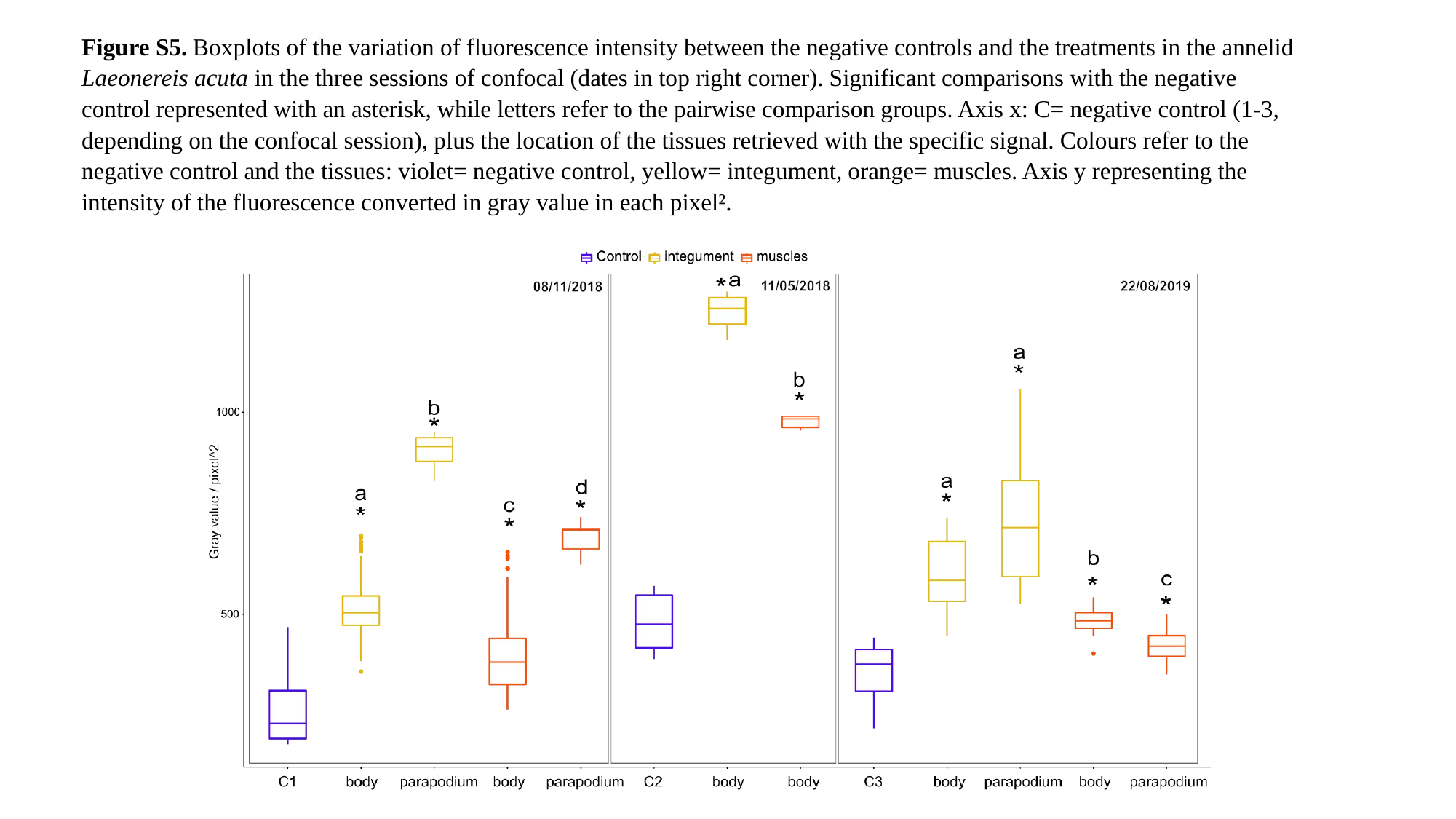

Figure S5. Boxplots of the variation of fluorescence intensity between the negative controls and the treatments in the annelid Laeonereis acuta in the three sessions of confocal (dates in top right corner). Significant comparisons with the negative control represented with an asterisk, while letters refer to the pairwise comparison groups. Axis x: C= negative control (1-3, depending on the confocal session), plus the location of the tissues retrieved with the specific signal. Colours refer to the negative control and the tissues: violet= negative control, yellow= integument, orange= muscles. Axis y representing the intensity of the fluorescence converted in gray value in each pixel².

## Slide 6
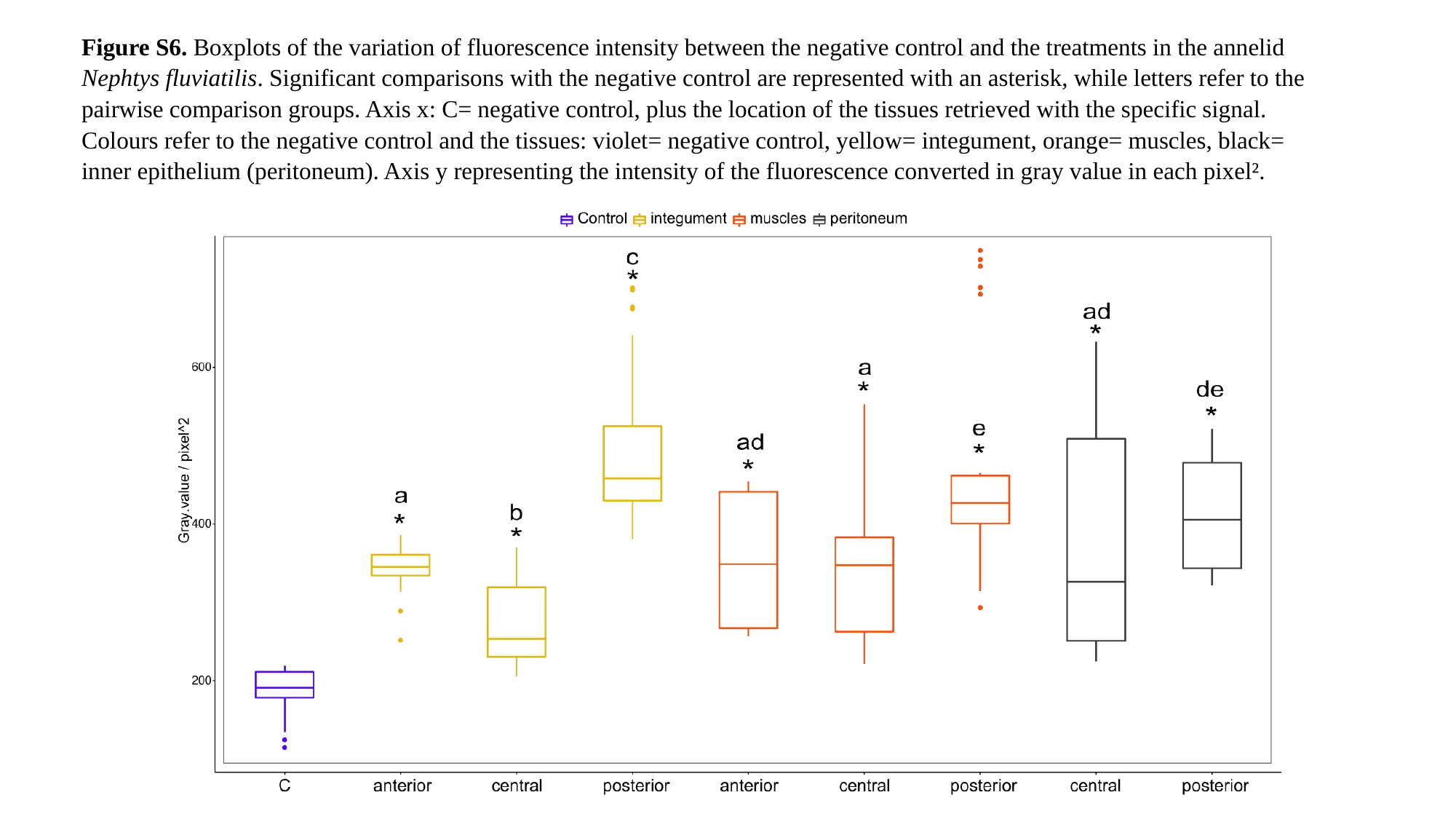

Figure S6. Boxplots of the variation of fluorescence intensity between the negative control and the treatments in the annelid Nephtys fluviatilis. Significant comparisons with the negative control are represented with an asterisk, while letters refer to the pairwise comparison groups. Axis x: C= negative control, plus the location of the tissues retrieved with the specific signal. Colours refer to the negative control and the tissues: violet= negative control, yellow= integument, orange= muscles, black= inner epithelium (peritoneum). Axis y representing the intensity of the fluorescence converted in gray value in each pixel².

## Slide 7
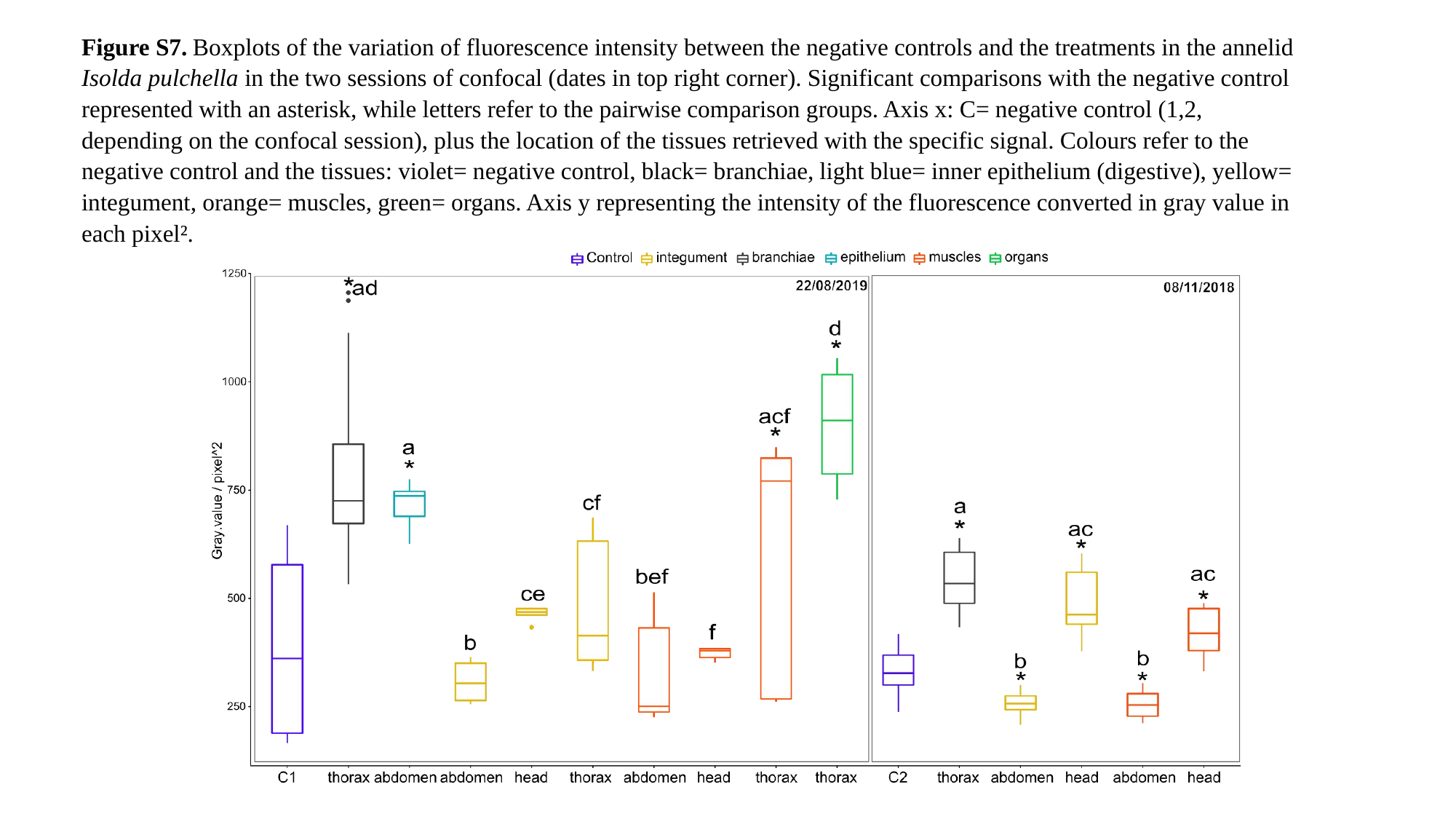

Figure S7. Boxplots of the variation of fluorescence intensity between the negative controls and the treatments in the annelid Isolda pulchella in the two sessions of confocal (dates in top right corner). Significant comparisons with the negative control represented with an asterisk, while letters refer to the pairwise comparison groups. Axis x: C= negative control (1,2, depending on the confocal session), plus the location of the tissues retrieved with the specific signal. Colours refer to the negative control and the tissues: violet= negative control, black= branchiae, light blue= inner epithelium (digestive), yellow= integument, orange= muscles, green= organs. Axis y representing the intensity of the fluorescence converted in gray value in each pixel².
